# Prevalence of antibodies against seasonal influenza A and B viruses among older adults in rural Thailand: A cross-sectional study

**DOI:** 10.1371/journal.pone.0256475

**Published:** 2021-08-30

**Authors:** Nungruthai Suntronwong, Preeyaporn Vichaiwattana, Lakkhana Wongsrisang, Sirapa Klinfueng, Sumeth Korkong, Thanunrat Thongmee, Nasamon Wanlapakorn, Yong Poovorawan

**Affiliations:** Faculty of Medicine, Department of Pediatrics, Center of Excellence in Clinical Virology, Chulalongkorn University, Bangkok, Thailand; Stanford University School of Medicine, UNITED STATES

## Abstract

Assessing the seroprevalence of the high-risk individuals against the influenza virus is essential to evaluate the progress of vaccine implementation programs and establish influenza virus interventions. Herein, we identified the pre-existing cross-protection of the circulating seasonal influenza viruses among the older-aged population. A cross-sectional study was performed base on the 176 residual sera samples collected from older adults aged 60 to 95 years without a history of vaccination in rural Thailand in 2015. Sera antibody titers against influenza A and B viruses circulating between 2016 and 2019 were determined by hemagglutination inhibition assay. These findings indicated the low titers of pre-existing antibodies to circulating influenza subtypes and showed age-independent antibody titers among the old adults. Moderate seropositive rates (HAI ≥ 1:40) were observed in influenza A viruses (65.9%A(H3N2), 50.0% for A(H1N1) pdm09), and found comparatively lower rates in influenza B viruses (14% B/Yam2, 21% B/Yam3 and 25% B/Vic). Only 5% of individuals possessed broadly protective antibodies against both seasonal influenza A and B virus in this region. Our findings highlighted the low pre-existing antibodies to circulating influenza strains in the following season observed in older adults. The serological study will help inform policy-makers for health care planning and guide control measures concerning vaccination programs.

## Introduction

The influenza virus is a significant pathogen that causes respiratory tract infections resulting in substantial morbidity and mortality during the annual influenza epidemic. A previous report indicated that influenza-associated deaths were estimated globally to be 290,000 to 650,000 individuals each year [1]. Influenza vaccination is a primary tool to effectively prevent influenza infection and reduce severe influenza-related sequelae [2]. The Advisory Committee on Immunization Practices (ACIP) recommends influenza vaccination of individuals at risk for severe complications, including pregnant women, young children (6–59 months), older adults, individuals with chronic medical conditions, and health care personnel [3].

Among the high-risk groups, the highest influenza-related mortality rate typically occurs among individuals older than 65 years of age (2.9 to 44 per 100,000 individuals for persons aged 65–74 years and 17.9 to 223.5 per 100,000 individuals for people aged over 75 years) [4]. Increases in influenza-associated hospitalization and death in this age group occur most frequently in individuals with co-morbidities and immunosuppression [5–7]. With advanced age, a meta-analysis indicated that vaccine effectiveness (VE) against influenza-like illness (ILI) and laboratory-confirmed influenza were estimated as approximately 39% and 49%, respectively, in older adults [8]. This result is most likely due to immunosenescence, which is a major contributing factor to increasing infection susceptibility and directly leads to a decline in the immune response, both humoral and cell-mediated immunity [9]. Although older adults could not maintain the seroprotective titers to the vaccine strain and most of these levels likely wane prior to the next season; individuals receiving the influenza vaccine might have a lower risk of influenza infection (from 6% to 2.4%) than an individual who has not been vaccinated [10]. Therefore, monitoring the immunity status of influenza viruses should be considered in older adults.

The older-aged population (≥ 60 years of age) has increased by 1.2% in Thailand, from 10.7 million in 2015 to 13 million in 2020 [11]. Vaccination with the annual influenza vaccine in individuals aged more than 65 has been recommended since 2008 to reduce the influenza burden [12,13]. Several studies have reported the seroprevalence rate among high-risk groups such as young children, health care workers, individuals with chronic medical conditions, and pregnant women [14–16]. Data of seroprevalence is valuable in pandemic scenarios and helps determine pre-existing immunity and reflect vaccine coverage. However, the seroprevalence data of the older-aged population are lacking. Thus, we aim to assess existing antibodies against seasonal influenza A/H1N1pdm09, A/H3N2, and B viruses to determine the seropositive rates in individuals aged older than 60 years.

## Materials and methods

### Ethical approval

The Institutional Review Board approved the study protocol, which was conducted according to the principles of the Declaration of Helsinki and the Good Clinical Practice Guidelines (ICH-GCP) of the Faculty of Medicine of Chulalongkorn University (IRB No. 127/61). The IRB waived the need for consent as the study on anonymized residual samples.

### Serum samples

In our study, 176 residual sera (~200 μL) from a cross-sectional descriptive study performed in the rural northeastern region of Thailand (Chum Phae, Khon Kaen province) from October to November 2015 were obtained [17]. Serum samples were collected from older adults aged ≥60 years. Serological testing against influenza A/H1N1pdm09, A/H3N2, and influenza B viruses were performed for individual serum samples. Data regarding the date of vaccination and the date of the previous infection were not recorded.

### Representative influenza viruses

To identify the presence of anti-influenza antibodies, we selected the representative influenza A/H1N1pdm09, A/H3N2, B/Victoria, and B/Yamagata viruses that circulated during the 2017–2019 influenza season as the test viruses. The nasopharyngeal swabs tested positive for influenza A/H1N1pdm09, and A/H3N2 were propagated in 80% confluent Madin-Darby canine kidney (MDCK) cells [18] and incubated at 37°C for 3–5 days, while the influenza B virus was grown and incubated at 33°C for three days. The supernatants were collected and inoculated in embryonated chicken eggs that incubated at 37°C for 48 h. Eggs were then cooled to 4°C overnight, and the allantoic fluids were harvested and cleared by centrifugation. All viruses were subjected to conventional PCR and Sanger sequencing. The primer sets for both influenza A and B viruses have been described elsewhere [19,20].

The nucleotide sequences of the propagated viruses were assembled using Seq-Man Pro (DNASTAR, Madison, WI, USA) compared to the nucleotide sequences of the original samples using MUSCLE. Phylogenetic tree for each influenza type and subtype was constructed using the nucleotide sequences obtained from this study and southern hemisphere vaccines in 2015 available from the Global Initiative for Sharing All Influenza Data (GISAID) (http://platform.gisaid.org) database (S1 Fig). Trees were generated using the maximum-likelihood method and implemented in MEGAX [21]. The tested viruses were A/Thailand/CU-CN364/2017 (clade 6B.1) for A/H1N1pdm09 (accession No. MW600330) and A/Thailand/CU-B36461/2018 (clade 3C.3a) for A/H3N2 (accession No. MW600331). The B/Thailand/CU-B31196/2019 (accession No. MT803438), B/Massachusetts/02/2012_E1 (MW600489), and B/Thailand/CU-B26097/2018 (accession No. MT803450) were chosen as representative for the B/Victoria clade 1A, B/Yamagata clade 2, and B/Yamagata clade 3, respectively.

### Hemagglutinin inhibition assay

All sera were stored at -20°C and brought to room temperature before testing. Serum samples were quantitatively assessed to determine the antibody titer in duplicate using the hemagglutinin inhibition (HAI) assay, as described previously [22]. Individual serum samples were pre-treated with the receptor-destroying enzyme (RDE; Denka Seiken, Tokyo, Japan) at a dilution of 1:4 and then incubated at 37°C overnight to remove nonspecific inhibitors of hemagglutinin. Serum samples were further incubated at 56°C to inactivate the process, which was followed by adding phosphate-buffered saline to achieve a 1:10 dilution. RDE-treated sera were tested for nonspecific HA activity using 0.5% suspension turkey red blood cells. If agglutinin appeared, adsorption of sera with turkey red blood cells could be performed by incubating the packed RBCs, and RDE treated serum at 4°C for 1 h. Two-fold serial dilution (25 μL) of RDE-treated sera were prepared in a 96-well V-bottom plate starting at a 1:10 dilution and subsequently incubated with 25 μL of the tested virus (4 HA units per 25 μL) at room temperature for 30 min. The hemagglutination titer was read after adding a 0.5% suspension of turkey red blood cells and was incubated at 4°C for 1 h. The highest reciprocal of the serum dilution that completely inhibited hemagglutination was recorded as the endpoint titer. A HAI titer more than or equal to 40 was considered protective against the tested virus [23,24]. We compared the antibody level. A HAI titer less than 10 was assigned a value of 5 to calculate the geometric mean titer (GMT) as in previous studies [25,26].

### Statistical analysis

Seropositive rate was calculated using the number of individuals who were seropositive (considering only HAI titer ≥ 1:40) divided by the total number of subjects. The estimated data points for each influenza type or subtype were reported as a number, percentage, and 95% confidence intervals (CI). The differences between categorical variables (gender, age, and chronic disease) were evaluated by the chi-square test. The Dunn procedure was used as a posttest for pairwise comparisons of antibody titers among influenza types or subtypes following significant Friedman tests. The Spearman rank correlation between age and antibody titers for influenza virus was computed using R v3.6.0 (R Foundation for Statistical Computing, Vienna, Austria; https://www.r-project.org). All statistical analyses were performed using SPSS version 23 (IBM Corp., Armonk, NY, USA) and GraphPad Prism version 8.0.0 (GraphPad, San Diego, CA, USA; https://www.graphpad.com). The *p* value ≤ 0.05 was considered statistically significant.

## Results

### Patient characteristics

This study included 176 subjects, 21.6% (38/176) men and 78.4% (138/176) women. Their ages ranged from 60 to 95 years with a median of 69 (IQR:10.8) years. Approximately 48.86% (86/176) of this population presented at least one type of underlying disease, including diabetes mellitus with and without hypertension, were 18.6% (16/86) and 38.38% (33/86), respectively. Of 86, 40.7% of participants only presented hypertension and 1.16% combined hypertension and coronary heart disease. Moreover, 1.16% of participants showed diabetes mellitus combined with hypertension and coronary heart disease (S1 Table). Serum samples tested with the circulating influenza strains and the HAI-specific antibody titers are shown in S2 Table.

### Antibody titers against influenza A and B viruses

**Fig 1:**
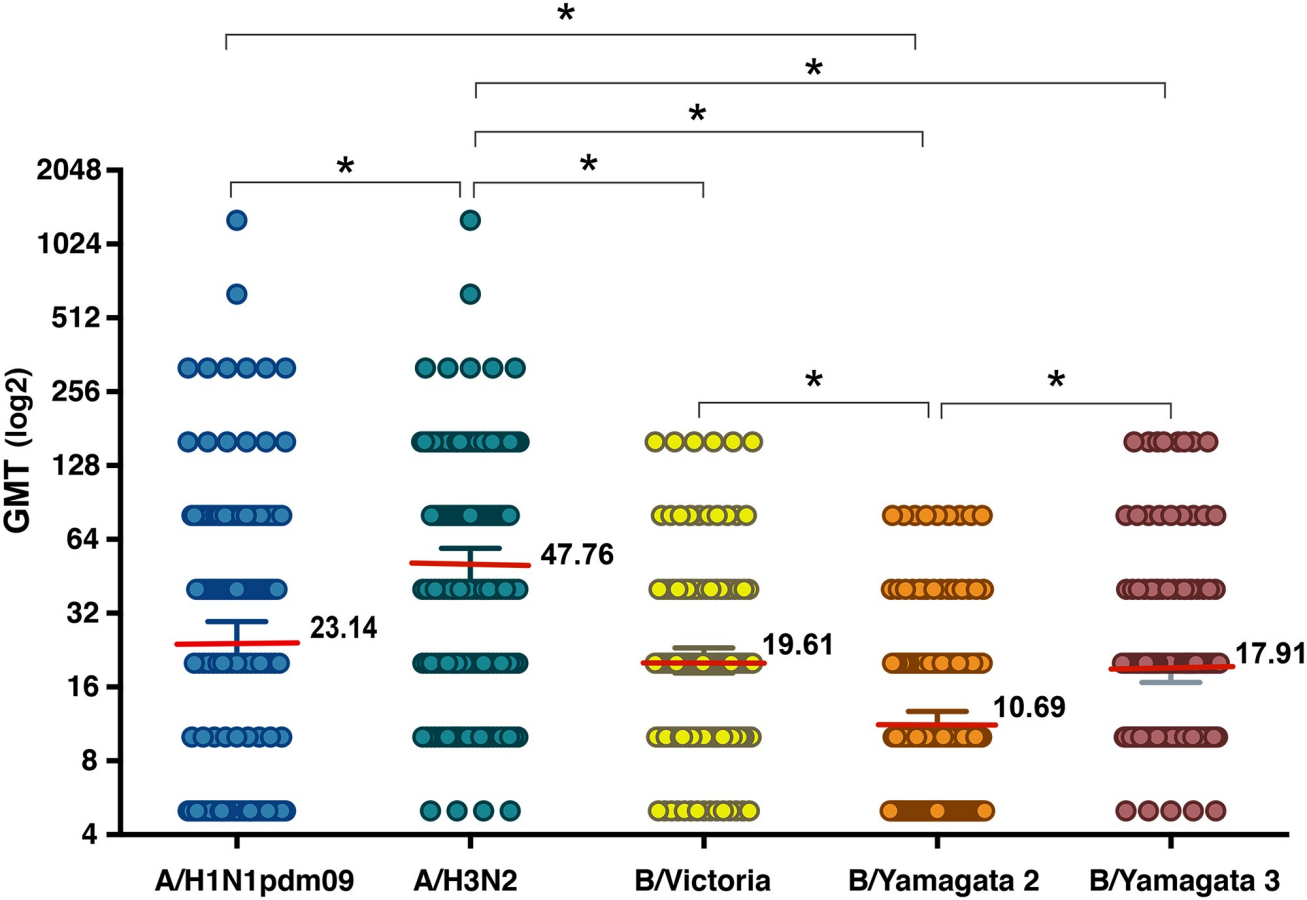
Comparison of the geometric mean titers among influenza type or subtype in the older adults. The colored circles are defined as influenza virus: A) A/H1N1pdm09 (A/Thailand/CU-CN364/2017, purple), B) A/H3N2 (A/Thailand/CU-B36461/2018, yellow), C) B/Victoria (B/Thailand/CU-B31196/2019, green), D) B/Yamagata 2 (B/Massachusetts/02/2012, blue) and E) B/Yamagata 3 (B/Thailand/CU-B26097/2018, pink). The Y-axis indicates the geometric mean titer (GMT), and the X-axis showed influenza type or subtype. The red line indicates the GMT for individual influenza virus. Error bars revealed 95% confidence intervals (95% CI). The asterisk refers to a significance level <0.0001.

Among the 176 tested samples, the geometric mean titers among influenza A subtypes in the elderly indicated that the highest antibody level was 47.8 (95% CI: 40.6–56.2), which was detected against influenza A/H3N2 (Table 1). While the HAI-specific antibodies of influenza A/H1N1pdm09 were 23.1 (95% CI: 19.0–28.2), which was significantly lower than that of influenza A/H3N2 (p<0.001) (Fig 1). Among influenza B viruses, the geometric mean titer of influenza B/Victoria (19.6, 95% CI: 17.4–22.1) and B/Yamagata 3 (17.9, 95% CI: 15.9–20.1) were significantly higher than that of the B/Yamagata 2 lineage (10.7, 95% CI: 9.4–12.1) (p<0.001). Furthermore, the HAI-specific antibody titers to both influenza A subtypes were slightly higher than those of the influenza B virus in this study population.

**Table 1:** Geometric means titers against seasonal influenza virus and seroprevalence among the elderly population.

Influenza virus	^a^HAI titer ≥ 1:40		^b^GMT		
	Number/Total	%	Mean	95%CI	
Influenza A virus					
A/H1N1pdm09	88/176	50.00	23.14	18.99	28.19
A/H3N2	116/176	65.91	47.76	40.56	56.23
influenza B virus					
B/Victoria	44/176	25.00	19.61	17.42	22.08
B/Yamagata2	25/176	14.20	10.69	9.42	12.13
B/Yamagata 3	37/176	21.02	17.91	15.94	20.13

^a^ HAI, hemagglutination inhibition;

^b^ GMT, Geometric means titers.

**Fig 2:**
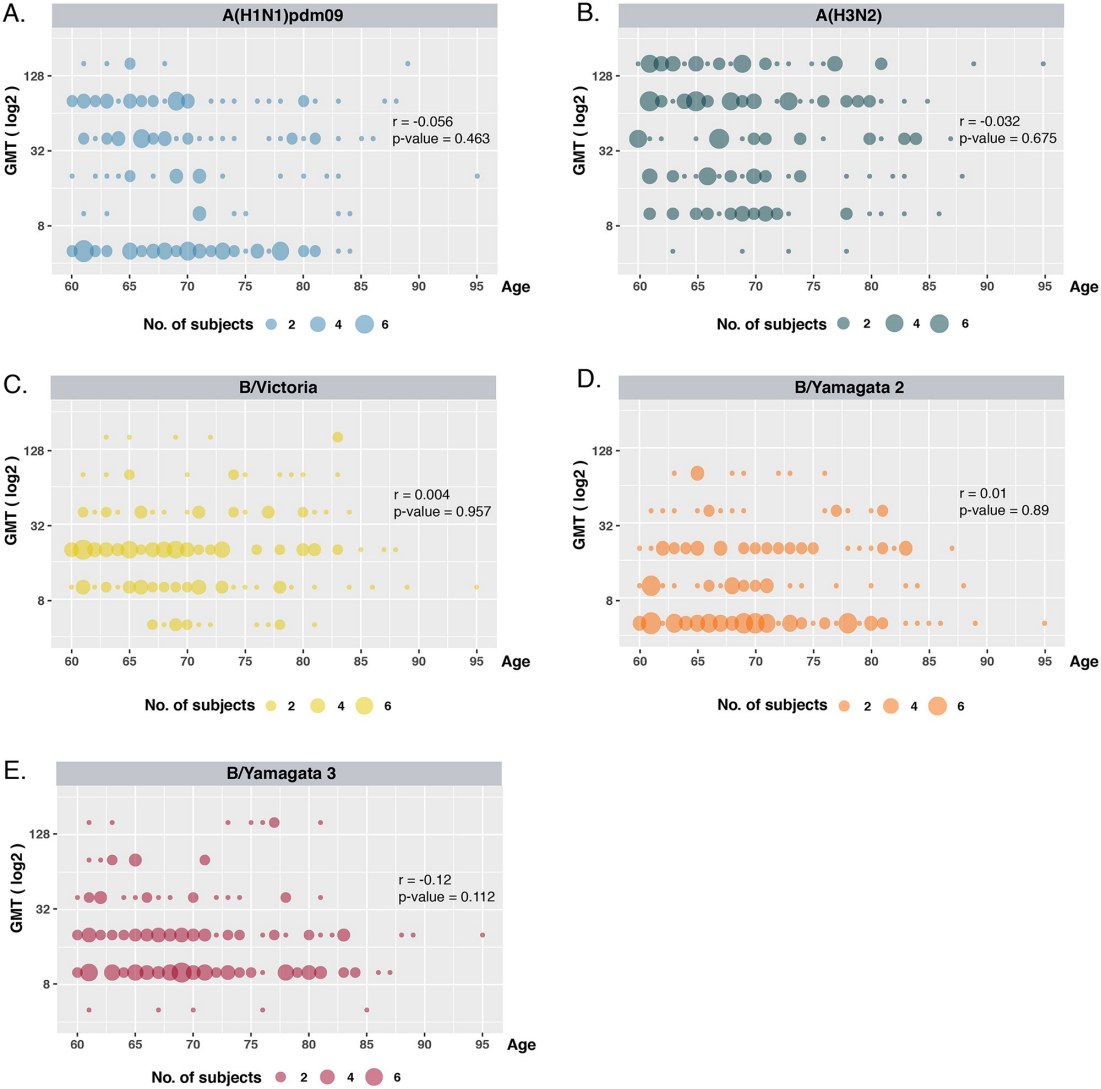
Correlation between age and antibody titers for influenza A and B viruses among the elderly. The Spearman rank correlation was conducted to identify the relationship between the age of individuals and the geometric mean titers (GMT) for individual influenza type or subtype. (A) influenza A/H1N1pdm09(blue); (B) influenza A/H3N2 (green); (C) influenza B/Victoria (yellow); (D) influenza B/Yamagata 2(orange) and (D) influenza B/Yamagata 3 (pink) are indicated. The X-axis shows the age range from 60 to 95 years old. The Y-axis indicates the geometric mean titers (GMT) with a logarithm scale. The size of the circles corresponds to the number of subjects.

We analyzed associations between age and geometric mean titers (GMT). The results showed no significant correlation between age and the antibody titers to substantial influenza strains in the next season (Fig 2). It suggested that the antibody titers were age-independent among the older adults.

### Seroprevalence of influenza A and B virus among the older adults

Overall, a higher seroprevalence against influenza A virus than influenza B virus was observed (Table 1). The highest seropositive rates were found in individuals who were possess seropositivity for influenza A/H3N2 antibodies, which accounted for 65.91% (116/176). The seropositive rates for influenza A/H1N1pdm09 were subsequently estimated to be 50% (88/176). Although the seroprevalence of either influenza A/H1N1pdm09 or A/H3N2 antibodies were estimated to be approximately half that of the study populations, the individuals who tested seropositive for both influenza A subtypes comprised 29.5% (52/176). Unlike the influenza A virus, the proportion of individuals who tested positive for influenza B antibodies was low. The seropositive rates did not significantly differ between B/Victoria (25%) and B/Yamagata 3 (21%). Approximately 12% (21/176) of the elderly possessed antibodies against B/Victoria and B/Yamagata lineages.

**Fig 3:**
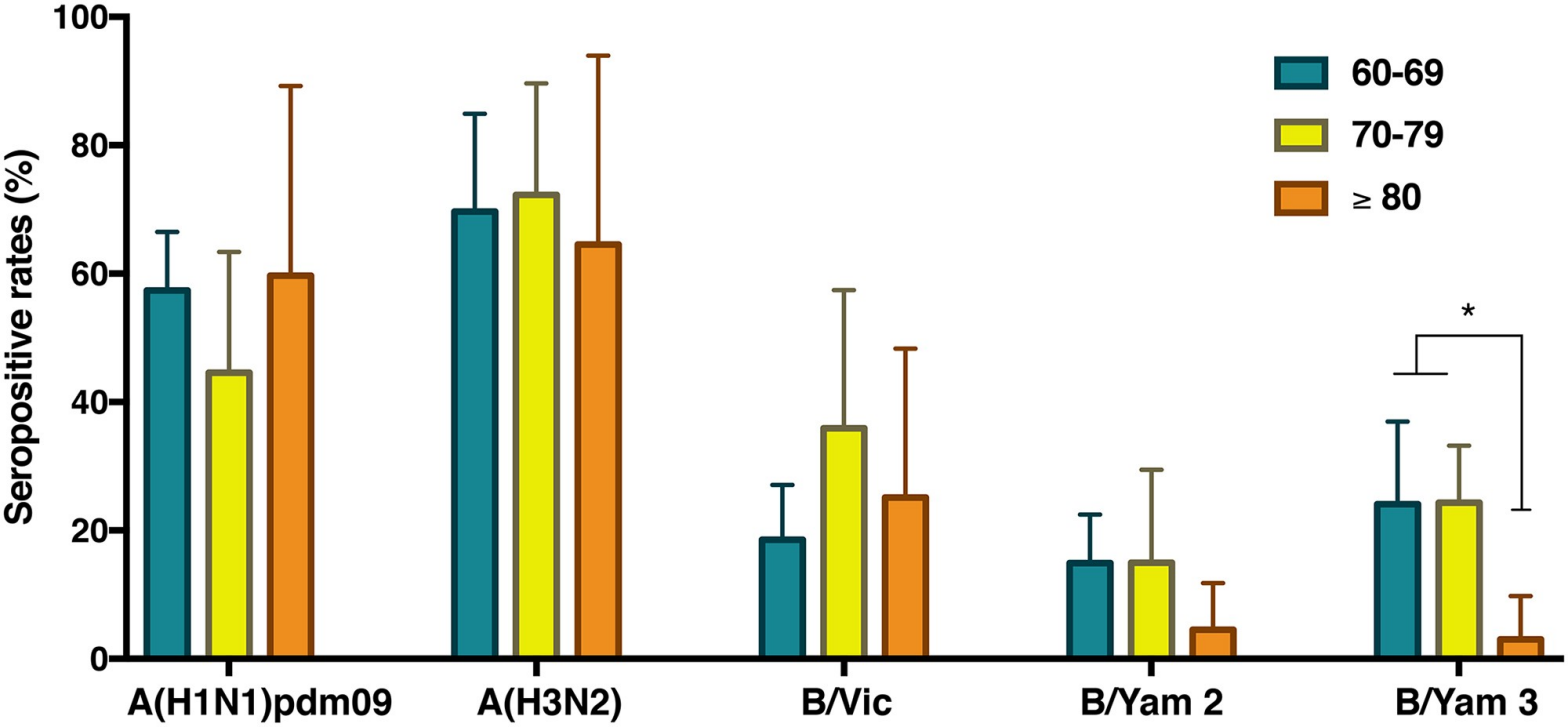
Age group-specific seroprevalence against seasonal influenza A and B virus in elderly individuals (aged 60–95 years) in rural Thailand, 2015. The bar graphs represent the seropositive rates (Y-axis) relative to individual influenza type or subtype (X-axis). The stratified age groups were defined by colors as: 60–69 (green), 70–79 (yellow), and older than 80 (orange) years of age. The error bars present the 95% confident intervals for the point of estimates. The asterisk refers to the significance level <0.01 (Kruskal-Wallis test).

In the analysis stratified by age groups, most individuals presented higher seropositive rates of influenza A virus than influenza B virus (Fig 3). Although there was no significant difference in seroprevalence rates among the age groups for influenza A virus, individuals were more likely to possess more antibodies against influenza A/H3N2 than influenza A/H1N1pdm09 (S3 Table). The lowest seroprevalence was noticed for the B/Yamagata lineages, which appeared to decrease dramatically in individuals older than 80 years of age (Y2: p = 0.068, Y3: p = 0.002). However, there were no statistically significant differences in seropositive rates based on gender or chronic diseases (S4 Table).

### Low seropositive rates for all influenza types or subtypes

**Fig 4:**
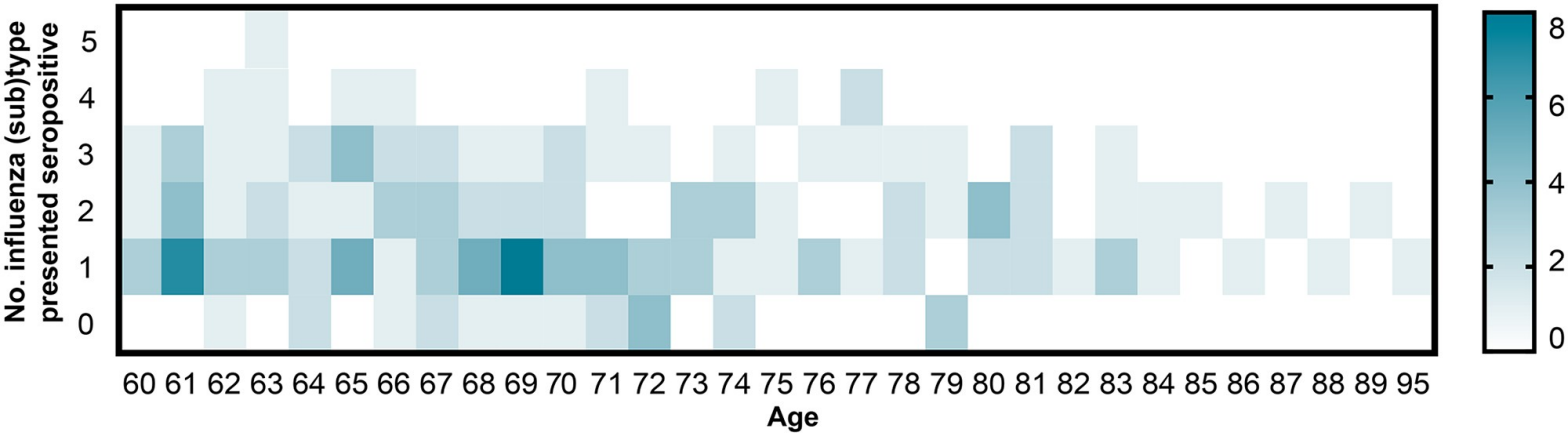
Schematic representation of the number of influenza type or subtype presenting seropositivity (HAI titer ≥ 1:40) for the individual. The X-axis indicates the age range of the elderly, and the Y-axis represents the number of seropositive influenza type or subtype. Colored intensity indicated the number of individuals.

To identify the proportion of individuals who presented broad protective antibodies, we determined the number of individuals who were seropositive against at least one influenza type or subtype. Overall, 89% (156/176) of the elderly individuals in our study possessed antibodies against at least one influenza type or subtype, while 11% (20/176) of individuals was seronegative (Fig 4). The heterologous immunity of different influenza B lineage was relatively lower (11.93%) than different influenza A subtypes (30.68%). Although most individuals (42%) were seropositive to one influenza type or subtype, approximately 5% of this population possessed the antibodies to four influenza types or subtypes which indicated a small proportion of individuals provided broadly protective antibodies to all seasonal influenza viruses.

## Discussion

Herein, we identified the prevalence of pre-existing antibodies against the seasonal influenza viruses among the older aged population in a rural area of Thailand. Our study found that most antibodies against influenza A viruses were moderate in aging adults, while the antibodies against influenza B viruses were comparatively lower. There was an age-independent antibody titer found in individuals older than 60 years of age. Further, a small proportion of the elderly possessed broadly seroprotective antibodies to all seasonal influenza viruses in this study.

A moderate protective level of antibodies to the influenza A virus might occur because of the seasonal epidemic of the influenza A virus [27]. A newly antigenic drift virus was introduced, resulting in the typical influenza epidemic. Exposure to the drifted influenza virus was able to increase or maintain pre-existing immunity [28]. Moreover, time since infection may be related to different titers of HAI-specific antibodies. Since influenza A(H1N1)pdm09 emerged in 2009, the incidence was peak dramatically from 2010 until mid-2011 [29]. Although influenza A(H1N1)pdm09 remains circulated since then, the predominant subtype was influenza A (H3N2) [27]. Typically, the level of antibody detection decrease with time since exposure to the historical strains [30]; it was probably related to the antibody titers to A(H1N1) pdm09 comparatively lower than A(H3N2) virus. Furthermore, the highest titer of HAI-specific antibodies to A/H3N2 was consistent with the predominant subtype of the influenza A virus, A/H3N2, in the 2015 influenza season in Thailand (June to August) [31].

A low protective level against influenza B virus might be related to the annually sporadic circulating activity compatible with influenza A virus [27]. According to influenza surveillance indicated that the B/Victoria-lineage was predominant between 2010–2012, while B/Yamagata lineage (clade 2) was significantly distributed in 2013 until the first half of 2014 and replaced by B/Yamagata (clade 3) since then [20,32]. It was consistent with our results indicated the antibody titers of B/Yamagata clade 3 comparatively higher than B/Yamagata clade 2. Although B/Victoria was less predominant in 2013, the antibody to B/Victoria remains higher presented in older adults than B/Yamagata clade 3. In addition, the evolution rate of influenza B hemagglutinin, the main antigenic determinant protein, is substantially lower than for the influenza A virus [33]. The higher seropositive rates to influenza B/Victoria than to the influenza B/Yamagata are consistent with a previous study from Spain [34]. However, the heterologous of different influenza B lineages was observed, which may relate to the lineage shift evidence between 2014–2016 [32]. Furthermore, the fewer individuals who possessed antibodies to those viruses might be related to the inferior persistence of antibodies against the influenza B virus compared to the influenza A virus [35].

The seroprotection was defined as HAI titer ≥ 1:40 that typically corresponds to a 50% protection against influenza infection in the elderly when the virus matched the circulating strains; in contrast, a higher titer (203–437) was required to achieve the mismatched viruses [36]. Our study found that most individuals did not show high titers against influenza A and B viruses, which may result from individuals who have never been vaccinated or from individuals whose immunity has waned [9]. The previous report found that the pre-existing immunity to the influenza virus affects the rate of increasing seroconversion in both younger and older people and can raise the breadth of immune responses following influenza vaccination [30,37]. However, there is evidence that HI titers in older adults do not reliably persist year-round and more likely to decline prior to the next season [37,38].

Vaccination of the elderly is a practical approach to reduce the likelihood of experiencing disease and death caused by influenza virus infection [8]. According to influenza vaccination guidelines [39], the authorities recommend that vaccine coverage for individuals older than 65 against seasonal influenza should exceed 75%. An evaluation of influenza vaccination following its introduction in Thailand suggests that the number of vaccines purchased increased by 25% [40]. Although a previous report estimated that the vaccine coverage among the elderly increased from 12% (2010) to 20% (2016), a smaller proportion of individuals who showed broad protection to all influenza types or subtypes remained small in this study [41]. It reflects a lower vaccine uptake in the elderly and suggests the need for proactive public health intervention and preventive measures.

There are some limitations to this study. There is a lack of information regarding the previous history of influenza infections and the date of individual vaccinations. It was hard to interpret the source of influenza immunity, whether it be due to immunization or natural exposure. The results from the hemagglutination inhibition assay do not indicate the levels of all protective antibody including anti-hemagglutinin stalk antibodies and the anti-neuraminidase antibodies. Nevertheless, our study did not perform the assay that measured the functional antibodies such as neutralization assays or ELISA. Tests for cellular immunity were not performed in this study, although these may also be associated with protection [42]. Furthermore, the tested viruses used in this study lacked A/H3N2 clade 3C.2a, which has been the predominant circulating strain since 2016. Thus, we may have underestimated the cross-reactivity against A/H3N2.

As Thailand continues to increase influenza vaccination among high-risk groups, the continued monitoring of the influenza immunity profile among those populations should be performed [12,13]. Our findings indicated that fewer individuals possessed antibodies against seasonal influenza virus among the older aged population and highlight the need to improve the vaccine policies in rural areas of Thailand. A serological study of high-risk individuals will be necessary to assess vaccination progress and reflect the need for further public health intervention to combat influenza. This study emphasizes the need to raise concerns among vaccine policy-makers for high-risk populations.

## Supporting information

S1 Fig Schematic showed the phylogenetic trees of representative tested virus compared with their vaccine strain. Phylogenetic analyses of the nucleotide sequences of HA coding region of (A) influenza A/H1N1pdm09, (B) influenza A/H3N2, (C) B/Victoria, (D) B/Yamagata were compared with their vaccine strains and reference strains of known clades which available from the database. Color dot indicates the tested virus that used in this study.
(TIF)

S1 Table Demographic of participants. (DOCX)

S2 Table Hemagglutination inhibition titers (HAI titers) against the individual seasonal influenza type or subtype among the older adults in rural, Thailand. (DOCX)

S3 Table Comparison the seropositive rates (HAI titers ≥ 1:40) against seasonal influenza viruses among different aged groups. (DOCX)

S4 Table Comparison the seropositive rates (HAI titers ≥ 1:40) against seasonal influenza viruses by gender, age groups and individuals with or without chronic disease. (DOCX)
