# Supplementary figures and images for: Prevalence of antibodies against seasonal influenza A and B viruses among older adults in rural Thailand: A cross-sectional study
Source: PLoS One. 2021 Aug 30;16(8):e0256475. doi: 10.1371/journal.pone.0256475 (PMC8404998; doi:10.1371/journal.pone.0256475)

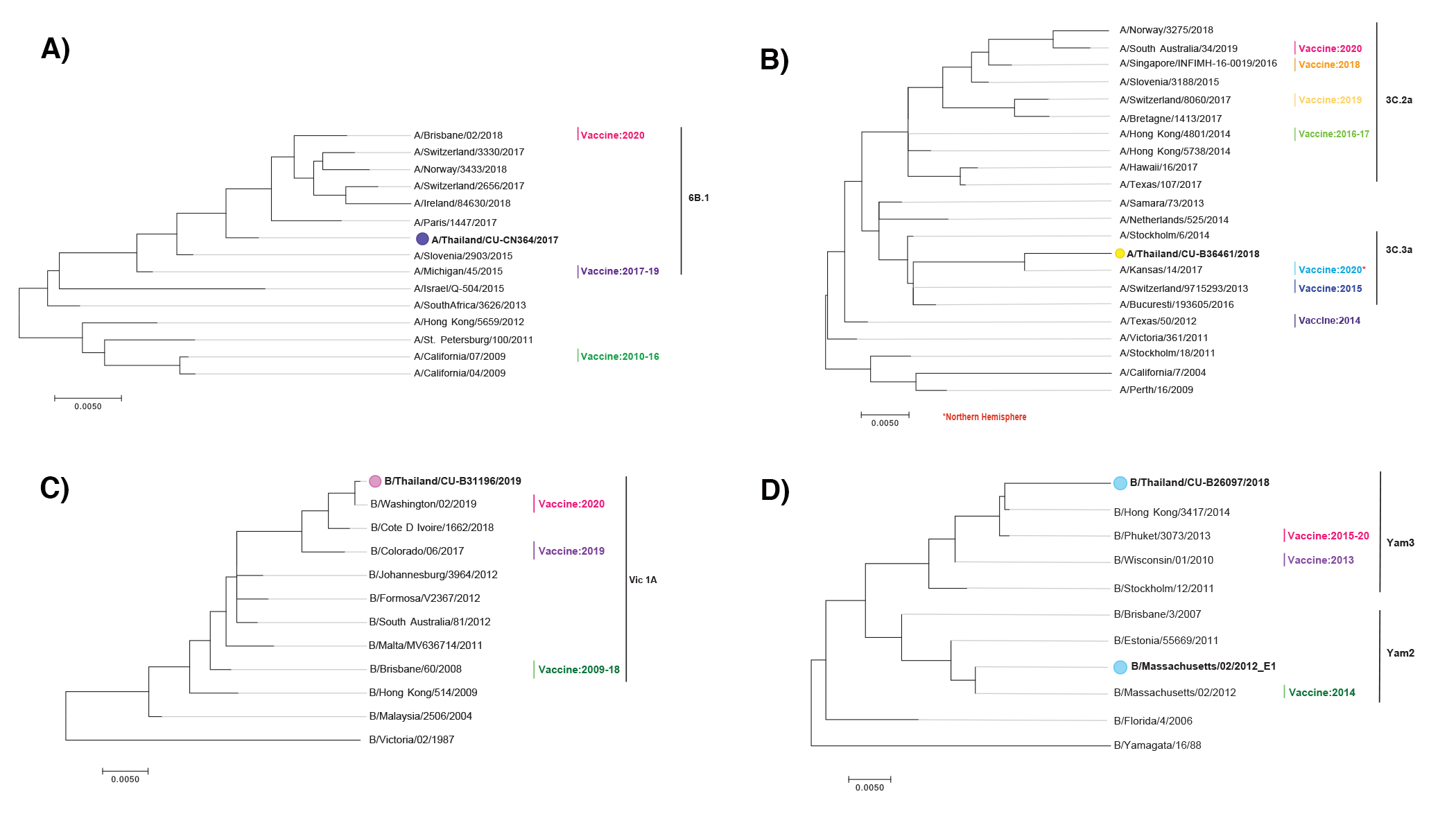

Supplement: S1 Fig — Phylogenetic analyses of the nucleotide sequences of HA coding region of (A) influenza A/H1N1pdm09, (B) influenza A/H3N2, (C) B/Victoria, (D) B/Yamagata were compared with their vaccine strains and reference strains of known clades which available from the database. Color dot indicates the tested virus that used in this study. (TIF) [file pone.0256475.s001.tif]
