# Supplementary material for: Prevalence of antibodies against seasonal influenza A and B viruses among older adults in rural Thailand: A cross-sectional study
Source: PLoS One. 2021 Aug 30;16(8):e0256475. doi: 10.1371/journal.pone.0256475 (PMC8404998; doi:10.1371/journal.pone.0256475)
Supplement: S1 Table — (DOCX) [file pone.0256475.s002.docx]

**S1 Table**. Demographic of participants.

| **Characteristics** | **Number (n=176)** | **%** | **Median (IQR)** |
| --- | --- | --- | --- |
| **Age, in years** |  |  | 69 (10.8) |
|  |  |  |  |
| **Sex** |  |  |  |
| Female | 138 | 78.41 |  |
| Male | 38 | 21.59 |  |
|  |  |  |  |
| **Underlying disease (n=86)** |  |  |  |
| DM | 16 | 18.60 |  |
| HT | 35 | 40.70 |  |
| DM+HT | 33 | 38.38 |  |
| HT+CHD | 1 | 1.16 |  |
| DM+HT+CHD | 1 | 1.16 |  |
|  |  |  |  |

DM=Diabetes mellitus; HT=Hypertension ; CHD =Coronary heart disease
