# Supplementary material for: Prevalence of antibodies against seasonal influenza A and B viruses among older adults in rural Thailand: A cross-sectional study
Source: PLoS One. 2021 Aug 30;16(8):e0256475. doi: 10.1371/journal.pone.0256475 (PMC8404998; doi:10.1371/journal.pone.0256475)
Supplement: S2 Table — (DOCX) [file pone.0256475.s003.docx]

**S2 Table. Hemaggl­­­utination inhibition titers (HAI titers) against the individual seasonal influenza (sub)type among the older adults in rural, Thailand.­­­­­­­**

| **ID** | **Age** | **A/H1N1p­­­­­dm09** | **A/H3N2** | **B/Vic** | **B/Yam2** | **B/Yam3** |
| --- | --- | --- | --- | --- | --- | --- |
|  |  | **A/Thailand/** | **A/Thailand/** | **B/Thailand/** | **B/Massachusetts** | **B/Thailand/** |
|  |  | **CU-CN364/2017** | **CU-B36461/2018** | **CU-B31196/2019** | **/02/2012** | **CU-B26097/2018** |
| 1 | 69 | 20 | 40 | 20 | 10 | 10 |
| 2 | 70 | 40 | 40 | 20 | 10 | 10 |
| 3 | 68 | <10 | 20 | 20 | 10 | 10 |
| 4 | 67 | <10 | 10 | <10 | <10 | 10 |
| 5 | 69 | 80 | <10 | 20 | 20 | 10 |
| 6 | 62 | 80 | 160 | 20 | 40 | 40 |
| 7 | 73 | 80 | <10 | 20 | 20 | 20 |
| 8 | 63 | 10 | <10 | 20 | <10 | 10 |
| 9 | 74 | 40 | 20 | 40 | 20 | 40 |
| 10 | 70 | 80 | 40 | 80 | <10 | 20 |
| 11 | 72 | 80 | 10 | 160 | 80 | 40 |
| 12 | 67 | 40 | 40 | <10 | 20 | 20 |
| 13 | 81 | 80 | 10 | 20 | 20 | 10 |
| 14 | 65 | 80 | 80 | 20 | 80 | 20 |
| 15 | 66 | 40 | 20 | 20 | 40 | 20 |
| 16 | 80 | 80 | 20 | 40 | <10 | 10 |
| 17 | 63 | 160 | 10 | 40 | 10 | 10 |
| 18 | 63 | 80 | 20 | 20 | <10 | 10 |
| 19 | 69 | 80 | 10 | 20 | 10 | 10 |
| 20 | 71 | 10 | 20 | 20 | 10 | 10 |
| 21 | 71 | 10 | 10 | 40 | 10 | 20 |
| 22 | 72 | 40 | 10 | 20 | 20 | 10 |
| 23 | 68 | 40 | 10 | 20 | 10 | 20 |
| 24 | 62 | 80 | 80 | 20 | 20 | 40 |
| 25 | 61 | 80 | 10 | 20 | <10 | 10 |
| 26 | 78 | 40 | 20 | 20 | <10 | 10 |
| 27 | 66 | 40 | 10 | 20 | <10 | 10 |
| 28 | 64 | 80 | 20 | 20 | <10 | 10 |
| 29 | 61 | 320 | 20 | 20 | 10 | 20 |
| 30 | 63 | 40 | 10 | 20 | 20 | 20 |
| 31 | 64 | 40 | 80 | 40 | <10 | 10 |
| 32 | 67 | 80 | 40 | 20 | 20 | 10 |
| 33 | 69 | 40 | 10 | <10 | <10 | 10 |
| 34 | 76 | 80 | 160 | <10 | 80 | 160 |
| 35 | 68 | 40 | 10 | 20 | 40 | 40 |

| **ID** | **Age** | **A/H1N1p­­­­­dm09** | **A/H3N2** | **B/Vic** | **B/Yam2** | **B/Yam3** |
| --- | --- | --- | --- | --- | --- | --- |
|  |  | **A/Thailand/** | **A/Thailand/** | **B/Thailand/** | **B/Massachusetts** | **B/Thailand/** |
|  |  | **CU-CN364/2017** | **CU-B36461/2018** | **CU-B31196/2019** | **/02/2012** | **CU-B26097/2018** |
| 36 | 60 | 80 | 40 | 20 | 20 | 40 |
| 37 | 63 | 80 | 160 | 20 | <10 | 10 |
| 38 | 79 | 40 | 80 | 80 | 20 | 10 |
| 39 | 66 | 40 | 10 | 40 | 10 | 40 |
| 40 | 69 | 80 | 20 | 20 | 20 | 10 |
| 41 | 61 | 80 | 10 | 40 | 10 | 10 |
| 42 | 62 | 20 | 40 | 20 | 10 | 20 |
| 43 | 66 | 40 | 20 | 40 | 40 | 20 |
| 44 | 70 | 80 | 20 | 20 | 20 | 20 |
| 45 | 65 | 320 | 10 | 20 | 20 | 80 |
| 46 | 70 | 80 | 10 | 20 | <10 | 10 |
| 47 | 65 | 80 | 20 | 10 | <10 | 10 |
| 48 | 69 | <10 | 10 | <10 | <10 | 10 |
| 49 | 65 | 160 | 160 | 20 | 80 | 80 |
| 50 | 71 | 20 | 10 | 10 | <10 | 20 |
| 51 | 68 | <10 | 80 | 10 | <10 | 10 |
| 52 | 73 | <10 | 10 | 10 | 10 | 10 |
| 53 | 71 | 20 | 10 | 10 | <10 | 10 |
| 54 | 66 | 40 | 80 | 40 | 10 | 40 |
| 55 | 61 | 80 | 20 | 20 | <10 | 10 |
| 56 | 84 | 10 | 40 | 10 | <10 | 10 |
| 57 | 68 | 160 | 20 | 10 | <10 | 20 |
| 58 | 86 | 40 | 10 | 10 | <10 | 10 |
| 59 | 65 | 20 | 10 | 10 | <10 | 20 |
| 60 | 78 | 20 | 10 | 10 | <10 | 10 |
| 61 | 80 | 20 | 10 | 40 | <10 | 10 |
| 62 | 78 | <10 | 10 | <10 | <10 | 20 |
| 63 | 83 | 80 | 10 | 80 | 10 | 20 |
| 64 | 70 | <10 | 10 | 40 | 20 | 40 |
| 65 | 75 | 320 | 320 | 80 | 20 | 160 |
| 66 | 67 | 320 | 160 | 40 | <10 | 20 |
| 67 | 70 | <10 | 80 | <10 | <10 | 20 |
| 68 | 65 | 20 | 160 | 10 | <10 | 10 |
| 69 | 77 | <10 | 160 | <10 | <10 | 20 |
| 70 | 65 | 80 | 160 | 80 | 20 | 20 |
| 71 | 95 | 20 | 160 | 10 | <10 | 20 |
| 72 | 81 | <10 | 160 | 10 | <10 | 160 |

|  |  |  |  |  |  |  |
| --- | --- | --- | --- | --- | --- | --- |
| **ID** | **Age** | **A/H1N1p­­­­­dm09** | **A/H3N2** | **B/Vic** | **B/Yam2** | **B/Yam3** |
|  |  | **A/Thailand/** | **A/Thailand/** | **B/Thailand/** | **B/Massachusetts** | **B/Thailand/** |
|  |  | **CU-CN364/2017** | **CU-B36461/2018** | **CU-B31196/2019** | **/02/2012** | **CU-B26097/2018** |
| 73 | 68 | 40 | 160 | 20 | 80 | 10 |
| 74 | 60 | <10 | 160 | 20 | <10 | 10 |
| 75 | 62 | <10 | 160 | 10 | <10 | 20 |
| 76 | 63 | <10 | 160 | 10 | <10 | 20 |
| 77 | 61 | 10 | 160 | 10 | 10 | 40 |
| 78 | 61 | 160 | 160 | 10 | <10 | 20 |
| 79 | 89 | 160 | 160 | 10 | <10 | 20 |
| 80 | 72 | <10 | 160 | <10 | <10 | 20 |
| 81 | 77 | 40 | 160 | 40 | 10 | 20 |
| 82 | 62 | <10 | 160 | 20 | 20 | 40 |
| 83 | 63 | 40 | 160 | 80 | 20 | 80 |
| 84 | 66 | 80 | 160 | 10 | <10 | 10 |
| 85 | 64 | 40 | 160 | 10 | <10 | 20 |
| 86 | 69 | 80 | 160 | 160 | 80 | 20 |
| 87 | 66 | <10 | 20 | 10 | <10 | 10 |
| 88 | 77 | 640 | 640 | 40 | 40 | 160 |
| 89 | 69 | 80 | 80 | 10 | <10 | 20 |
| 90 | 61 | <10 | 160 | 80 | 40 | 160 |
| 91 | 87 | 80 | 40 | 20 | 20 | 10 |
| 92 | 80 | <10 | 80 | 80 | 40 | 20 |
| 93 | 78 | <10 | 80 | 20 | 40 | 40 |
| 94 | 65 | <10 | 80 | 20 | 10 | 10 |
| 95 | 81 | <10 | 80 | <10 | <10 | 10 |
| 96 | 69 | <10 | 160 | 20 | <10 | 10 |
| 97 | 61 | <10 | 80 | 20 | <10 | 10 |
| 98 | 68 | <10 | 80 | <10 | <10 | 10 |
| 99 | 81 | 320 | 320 | 20 | 40 | 10 |
| 100 | 65 | <10 | 40 | 20 | 40 | 10 |
| 101 | 75 | 10 | 80 | 40 | 20 | 10 |
| 102 | 68 | <10 | 80 | 20 | 10 | 10 |
| 103 | 61 | <10 | 80 | 20 | 10 | 80 |
| 104 | 77 | 80 | 160 | 40 | 40 | 160 |
| 105 | 65 | <10 | 80 | 20 | 20 | 10 |
| 106 | 65 | <10 | 80 | 80 | <10 | 80 |
| 107 | 73 | 40 | 80 | 20 | 80 | 160 |
| 108 | 76 | <10 | 80 | 20 | 40 | 10 |
| 109 | 71 | <10 | 160 | 20 | 10 | 10 |

| **ID** | **Age** | **A/H1N1p­­­­­dm09** | **A/H3N2** | **B/Vic** | **B/Yam2** | **B/Yam3** |
| --- | --- | --- | --- | --- | --- | --- |
|  |  | **A/Thailand/** | **A/Thailand/** | **B/Thailand/** | **B/Massachusetts** | **B/Thailand/** |
|  |  | **CU-CN364/2017** | **CU-B36461/2018** | **CU-B31196/2019** | **/02/2012** | **CU-B26097/2018** |
| 110 | 71 | 40 | 160 | 40 | 20 | 80 |
| 111 | 83 | 40 | 80 | 160 | 20 | 20 |
| 112 | 81 | 40 | 160 | 20 | 20 | 40 |
| 113 | 64 | 20 | 80 | 20 | 20 | 20 |
| 114 | 67 | 80 | 160 | 10 | 20 | 20 |
| 115 | 73 | <10 | 80 | 10 | <10 | 20 |
| 116 | 73 | 1280 | 80 | 20 | <10 | 10 |
| 117 | 61 | <10 | 80 | 10 | <10 | 10 |
| 118 | 66 | 80 | 80 | 10 | <10 | 20 |
| 119 | 69 | 20 | 160 | 20 | 40 | 20 |
| 120 | 65 | 160 | 80 | 160 | 80 | 40 |
| 121 | 64 | 40 | 80 | 20 | 20 | 40 |
| 122 | 70 | 40 | 80 | 10 | 10 | 40 |
| 123 | 78 | <10 | 1280 | 80 | <10 | 10 |
| 124 | 60 | <10 | 40 | 20 | <10 | 20 |
| 125 | 76 | <10 | 40 | 10 | <10 | 20 |
| 126 | 61 | 40 | 160 | 40 | 10 | 20 |
| 127 | 67 | 20 | 40 | 20 | 10 | 20 |
| 128 | 61 | <10 | 80 | 20 | <10 | 20 |
| 129 | 69 | 20 | 80 | 10 | <10 | 10 |
| 130 | 73 | 20 | 160 | 20 | 20 | 40 |
| 131 | 70 | <10 | 20 | 10 | <10 | 10 |
| 132 | 69 | 320 | 160 | <10 | <10 | 20 |
| 133 | 74 | <10 | 80 | 40 | <10 | 10 |
| 134 | 71 | <10 | 40 | 10 | <10 | 10 |
| 135 | 62 | 40 | 80 | 40 | 20 | 80 |
| 136 | 81 | 40 | 40 | 40 | 40 | 20 |
| 137 | 78 | <10 | 320 | 10 | <10 | 10 |
| 138 | 83 | <10 | 40 | 20 | <10 | 10 |
| 139 | 83 | 20 | 40 | 20 | 20 | 20 |
| 140 | 71 | 20 | 40 | 40 | 20 | 80 |
| 141 | 80 | <10 | 40 | 20 | <10 | 10 |
| 142 | 70 | <10 | 20 | <10 | <10 | 10 |
| 143 | 75 | <10 | 160 | 10 | <10 | 10 |
| 144 | 73 | <10 | 20 | 10 | <10 | 10 |
| 145 | 61 | <10 | 80 | 20 | 10 | 10 |
| 146 | 73 | <10 | 80 | 20 | <10 | 10 |

| **ID** | **Age** | **A/H1N1p­­­­­dm09** | **A/H3N2** | **B/Vic** | **B/Yam2** | **B/Yam3** |
| --- | --- | --- | --- | --- | --- | --- |
|  |  | **A/Thailand/** | **A/Thailand/** | **B/Thailand/** | **B/Massachusetts** | **B/Thailand/** |
|  |  | **CU-CN364/2017** | **CU-B36461/2018** | **CU-B31196/2019** | **/02/2012** | **CU-B26097/2018** |
| 147 | 61 | 40 | 40 | 20 | 20 | 40 |
| 148 | 74 | 80 | 20 | 80 | 20 | 20 |
| 149 | 70 | <10 | 80 | 20 | <10 | <10 |
| 150 | 67 | <10 | 40 | 10 | <10 | <10 |
| 151 | 82 | 20 | 20 | 40 | 20 | 20 |
| 152 | 76 | <10 | 80 | 20 | <10 | <10 |
| 153 | 61 | <10 | 20 | 10 | <10 | <10 |
| 154 | 85 | 40 | 80 | 20 | <10 | <10 |
| 155 | 63 | 20 | 320 | 160 | 80 | 160 |
| 156 | 88 | 80 | 20 | 20 | 10 | 20 |
| 157 | 67 | 40 | 40 | 20 | <10 | 10 |
| 158 | 63 | <10 | 20 | 10 | <10 | 10 |
| 159 | 84 | <10 | 40 | 40 | 10 | 10 |
| 160 | 66 | <10 | 20 | 10 | <10 | 10 |
| 161 | 72 | <10 | 80 | 20 | 20 | 10 |
| 162 | 60 | 80 | 40 | 20 | 10 | 20 |
| 163 | 71 | <10 | 320 | <10 | <10 | 10 |
| 164 | 74 | 10 | 40 | 80 | <10 | 20 |
| 165 | 71 | 10 | 20 | 10 | <10 | 20 |
| 166 | 80 | 40 | 40 | 20 | 20 | 10 |
| 167 | 79 | 40 | 80 | 10 | <10 | 10 |
| 168 | 78 | <10 | <10 | <10 | <10 | 10 |
| 169 | 68 | 80 | 80 | 40 | 10 | 20 |
| 170 | 60 | 20 | 40 | 10 | <10 | 10 |
| 171 | 80 | 80 | 80 | 20 | 10 | 20 |
| 172 | 74 | <10 | 40 | 10 | 10 | 10 |
| 173 | 67 | <10 | 20 | 20 | 40 | 40 |
| 174 | 63 | 80 | 80 | 40 | 40 | 80 |
| 175 | 78 | 80 | 80 | 10 | 20 | 40 |
| 176 | 83 | 10 | 20 | 160 | 20 | 10 |
|  |  |  |  |  |  |  |
