# Supplementary material for: Prevalence of antibodies against seasonal influenza A and B viruses among older adults in rural Thailand: A cross-sectional study
Source: PLoS One. 2021 Aug 30;16(8):e0256475. doi: 10.1371/journal.pone.0256475 (PMC8404998; doi:10.1371/journal.pone.0256475)
Supplement: S3 Table — (DOCX) [file pone.0256475.s004.docx]

**S3 Table. Comparison the seropositive rates (HAI titers ≥ 1:40) against seasonal influenza viruses among different aged groups.**

|  |  | **HAI ≥ 1:40** | | **GMT** | | | **Kruskal-Wallis test** |
| --- | --- | --- | --- | --- | --- | --- | --- |
| **Virus** | **Age group** | **Number/ Total** | **%** | **Mean** | **Lower 95% CI Upper 95% CI** | | **p-value** |
|  |  |  |  |  |  |  |  |
| A/H1N1pdm09 | 60-69 | 52/92 | 56.52 | 29.37 | 22.83 | 37.79 | 0.261 |
|  | 70-79 | 22/58 | 37.93 | 19.79 | 14.22 | 27.56 |  |
|  | ≥80 | 14/26 | 53.85 | 35.52 | 23.56 | 53.54 |  |
| A/H3N2 | 60-69 | 61/92 | 66.30 | 47.93 | 38.58 | 59.54 | 0.937 |
|  | 70-79 | 37/58 | 63.79 | 49.01 | 35.39 | 67.88 |  |
|  | ≥80 | 18/26 | 69.23 | 44.5 | 30.02 | 65.96 |  |
| B/Victoria | 60-69 | 18/92 | 19.57 | 19.12 | 16.45 | 22.21 | 0.361 |
|  | 70-79 | 17/58 | 29.31 | 18.39 | 14.63 | 23.13 |  |
|  | ≥80 | 9/26 | 34.62 | 24.75 | 17.57 | 34.89 |  |
| B/Yamagata 2 | 60-69 | 15/92 | 16.30 | 10.95 | 9.124 | 13.13 | 0.068 |
|  | 70-79 | 7/58 | 12.07 | 10.24 | 8.188 | 12.81 |  |
|  | ≥80 | 3/26 | 11.54 | 10.83 | 7.945 | 14.77 |  |
| B/Yamagata 3 | 60-69 | 21/92 | 22.83 | 18.27 | 15.67 | 21.31 | 0.002 |
|  | 70-79 | 14/58 | 24.14 | 18.84 | 14.9 | 23.82 |  |
|  | ≥80 | 2/26 | 7.69 | 14.92 | 11.45 | 19.44 |  |
|  |  |  |  |  |  |  |  |
