# Supplementary material for: Prevalence of antibodies against seasonal influenza A and B viruses among older adults in rural Thailand: A cross-sectional study
Source: PLoS One. 2021 Aug 30;16(8):e0256475. doi: 10.1371/journal.pone.0256475 (PMC8404998; doi:10.1371/journal.pone.0256475)
Supplement: S4 Table — (DOCX) [file pone.0256475.s005.docx]

**S4 Table. Comparison the seropositive rates (HAI titers ≥ 1:40) against seasonal influenza viruses by gender and individuals with or without chronic disease.**

|  |  | **Gender** | | **Chronic disease** | |
| --- | --- | --- | --- | --- | --- |
|  |  | **Female (n=138)** | **Male (n=38)** | **No (n=91)** | **Yes (n=85)** |
| **Influenza A virus** |  |  |  |  |  |
| A/H1N1pdm09 | Number of (HAI ≥ 1:40)/ Total | 73/138 | 15/38 | 45/91 | 43/85 |
|  | Seropositivity (%) | **52.90** | **39.47** | **49.45** | **50.59** |
|  | p-value |  | 0.145 |  | 0.88 |
|  |  |  |  |  |  |
| A/H3N2 | Number of (HAI ≥ 1:40)/ Total | 87/138 | 29/38 | 64/91 | 52/85 |
|  | Seropositivity (%) | **63.04** | **76.32** | **70.33** | **61.18** |
|  | p-value |  | 0.13 |  | 0.202 |
|  |  |  |  |  |  |
| **influenza B virus** |  |  |  |  |  |
| B/Victoria | Number of (HAI ≥ 1:40)/ Total | 36/138 | 8 /38 | 25/91 | 19/85 |
|  | Seropositivity (%) | **26.09** | **21.05** | **27.47** | **22.35** |
|  | p-value |  | 0.527 |  | 0.434 |
|  |  |  |  |  |  |
| B/Yamagata 2 | Number of (HAI ≥ 1:40)/ Total | 20/138 | 5/38 | 13/91 | 12/85 |
|  | Seropositivity (%) | **14.49** | **13.16** | **14.29** | **14.12** |
|  | p-value |  | 0.835 |  | 0.975 |
|  |  |  |  |  |  |
| B/Yamagata 3 | Number of (HAI ≥ 1:40)/ Total | 29/138 | 8/38 | 17/91 | 20/85 |
|  | Seropositivity (%) | **21.01** | **21.05** | **18.68** | **23.53** |
|  | p-value |  | 0.996 |  | 0.431 |
|  |  |  |  |  |  |
